# Seabird Bycatch in Pelagic Longline Fisheries Is Grossly Underestimated when Using Only Haul Data

**DOI:** 10.1371/journal.pone.0012491

**Published:** 2010-08-31

**Authors:** Nigel Brothers, Alan R. Duckworth, Carl Safina, Eric L. Gilman

**Affiliations:** 1 Marine Ecology and Technology Consultant, Wonga Beach, Queensland, Australia; 2 Blue Ocean Institute, Cold Spring Harbor, New York, United States of America; 3 Tetra Tech EM Inc., and College of Natural and Computational Sciences, Hawaii Pacific University, Honolulu, Hawaii, United States of America; National Oceanic and Atmospheric Administration/National Marine Fisheries Service/Southwest Fisheries Science Center, United States of America

## Abstract

Hundreds of thousands of seabirds are killed each year as bycatch in longline fisheries. Seabirds are predominantly caught during line setting but bycatch is generally recorded during line hauling, many hours after birds are caught. Bird loss during this interval may lead to inaccurate bycatch information. In this 15 year study, seabird bycatch was recorded during both line setting and line hauling from four fishing regions: Indian Ocean, Southern Ocean, Coral Sea and central Pacific Ocean. Over 43,000 albatrosses, petrels and skuas representing over 25 species were counted during line setting of which almost 6,000 seabirds attempted to take the bait. Bait-taking interactions were placed into one of four categories. (i) The majority (57%) of bait-taking attempts were “unsuccessful” involving seabirds that did not take the bait nor get caught or hooked. (ii) One-third of attempts were “successful” with seabirds removing the bait while not getting caught. (iii) One-hundred and seventy-six seabirds (3% of attempts) were observed being “caught” during line setting, with three albatross species – Laysan (*Phoebastria immutabilis*), black-footed (*P. nigripes*) and black-browed (*Thalassarche melanophrys*)– dominating this category. However, of these, only 85 (48%) seabird carcasses were retrieved during line hauling. Most caught seabirds were hooked through the bill. (iv) The remainder of seabird-bait interactions (7%) was not clearly observed, but likely involved more “caught” seabirds. Bait taking attempts and percentage outcome (e.g. successful, caught) varied between seabird species and was not always related to species abundance around fishing vessels. Using only haul data to calculate seabird bycatch grossly underestimates actual bycatch levels, with the level of seabird bycatch from pelagic longline fishing possibly double what was previously thought.

## Introduction

Overexploitation of target species and high levels of bycatch, are the most widespread and direct causes of change and loss of global marine biodiversity [1]–[2]. Sea turtles, cetaceans, elasmobranchs and other fish species, and seabirds are particularly vulnerable to unsustainable levels of mortality and slow to recover from large population declines; bycatch in marine capture fisheries is putting some species in these groups at risk of extinction [3]–[7], risking permanent alteration to marine ecosystem functioning. On the order of hundreds of thousands of seabirds, including tens of thousands of albatrosses, are estimated to be caught annually in longline fisheries worldwide [8]–[9]. There is also evidence of high levels of seabird mortality in other marine capture fisheries, including trawl and gillnet fisheries [e.g., 10–12].

One of the major threats to some seabird populations is longline fishing [13], which involves a line up to 100 km in length carrying several thousand baited hooks. The target catch includes pelagic species like tunas, sharks and billfishes, and benthic species like toothfishes, halibut, and sablefish, but bycatch of unwanted fish, sea turtles and birds is often high. Seabird bycatch in longline fisheries occurs primarily in higher latitudes, where there is overlap with species of seabirds that interact with fishing vessels and are large enough to swallow a longline hook [14]–[15]. Of 61 species of seabirds affected by longline fisheries, 26 are threatened with extinction, including 18 of the 22 species of albatrosses, and there is compelling evidence that longline mortality is a significant component in the declines of many of these species [13]–[14], [16]–[17]. Albatross and large petrel species are particularly vulnerable because of their low fecundity, long sexual maturation and scavenging feeding behavior. Various gear technology mitigation methods, involving changes in fishing gear and methods, have been developed to reduce seabird bycatch, including bird-scaring ‘tori’ lines, underwater setting devices, side setting, blue-dyed bait, line weighting, night setting, and thawing bait, with varying degrees of efficacy at bird bycatch avoidance under experimental conditions [3], [14], [18]–[22]. Other approaches to mitigating bycatch in marine capture fisheries include input and output controls (measures to limit effort and catch), compensatory mitigation, marine spatial planning, fleet communication, and market-based mechanisms [7], [23]. These methods reduce the problem where they are required, there are large incentives for fishers to use them, and where enforcement is effective. But seabirds remain vulnerable in large regions.

In order to obtain more reliable estimates of seabird mortality levels in commercial fishing, which is essential to understand the effects of this threat [24], the present study compares seabird bycatch levels as determined from observations during line setting vs. line hauling from four fishing regions. Seabirds are mostly hooked when attempting to take the bait while the longline is being set, and subsequently drown [e.g., 20, 25–26]. Most previous studies counted captured birds not during line setting when the majority of birds are caught, but from the number of birds hauled aboard during gear retrieval. The present study explicitly compares the difference.

There has been evidence that this underestimates total bird capture [8], [13], [20], [25]. An estimated 27% of seabirds caught during setting by Japanese longline tuna vessels operating off of Tasmania, Australia, were not hauled aboard [8]. Gales et al. [13] observed crew from a Japanese longline southern bluefin tuna fishery and found that they discarded half (51%) of hooked seabirds by flicking or cutting them off branch lines while alongside the vessel; observers often fail to notice or record such birds. Gilman et al. [25] found that in the Hawaii longline tuna fishery 34% of seabirds caught during setting were not hauled aboard. In a subsequent study in the Hawaii longline tuna and swordfish fisheries, Gilman et al. [20] found that 28% of seabirds observed caught during setting were not hauled aboard. In the two Hawaii studies, crew did not attempt to dislodge or discard caught seabirds during hauling, and no birds were caught during gear hauling [20], [25]. In these studies, birds that had been observed hooked during gear setting but were not present upon gear retrieval can be inferred to have fallen from hooks due to scavenging, current, or other mechanical action during the line soak and haul [19]. Large differences between the number of birds observed caught on the set vs. the number hauled aboard has also been documented in other gear types: Of 30 seabirds observed captured during setting in a South African deep water trawl hake fishery, only two were hauled aboard [27].

## Methods

### Fishing Regions and Experimental Period

Seabird bycatch was observed by the lead author over a 15 year period, from 1988 to 2003, in four fishing regions: (i) Indian Ocean, mostly adjacent to the State of Western Australia; (ii) Southern Ocean, focusing around the State of Tasmania; (iii) Coral Sea, and areas northeast of Australia; and (iv) North-Central Pacific Ocean, an area located midway between Japan and the State of Hawaii. Data on seabird interactions were collected from 11 longline vessels during 305 sets, with most (84%) sets made during the day. Whenever logistically possible, observation of every hook set and recovered (hauled) on each day of fishing was maintained. Whenever maintaining observation of all of each set and haul was not possible, observation was made of the majority or all of the set but only a portion of the corresponding haul. In total, 781,307 baited hooks were deployed during these sets, of which 93% were observed being set and 67% were observed being hauled. This involved over 2,000 hours of observations. The last hook to be set was typically the first to be hauled, although occasionally a vessel would run back up a line to haul from the first hook that was set. This study incorporated datasets employed previously by Gilman et al. [20], [25] for the Hawaii longline tuna and swordfish fisheries, and pooled these with additional data not previously analyzed to produce a substantially larger sample size, where the previously employed data comprised 10% of the records employed here for the North-Central Pacific Ocean region.

Only data from pelagic longline fishing were used in this study because hooks are set at a lower rate relative to demersal longlining, about one hook every six seconds, allowing for accurate data collection. Demersal longliners, in contrast, set at the rate of five hooks per second, which does not allow sufficient time to obtain precise records of individual seabird interactions with the baited hook, especially when seabird abundance exceeds 100 birds astern.

Most seabird interaction data employed in this study were collected aboard fishing vessels that were using various measures to avoid catching birds. Brothers et al. [14], FAO [3] and Gilman et al. [20]–[22], [25] describe seabird bycatch mitigation methods.

### Bait-taking Categories

Seabird interaction observation methodology established in 1988 by Brothers [8] was used consistently throughout, a more detailed description of which is provided in Gilman et al. [25]. A seabird trying to take a bait off a hook was placed into one of the following categories: (i) successful, where it takes the bait and does not get caught; (ii) unsuccessful, where it fails to take the bait and does not get caught; (iii) caught, when the seabird gets caught or hooked; (iv) possibly caught, when it appears to get caught but this is not entirely clear; and (v) unsure, when the outcome is uncertain.

A bird sitting on the sea surface looking for baits underwater was not classified as a bait-taking attempt. When a seabird attempts to take the bait it makes a distinctive partial or total submerged body thrust towards it. Caught seabirds displayed clear evidence of struggle and inability to escape the line, such as persistent tugging whilst flapping backwards, making repeated attempts to fly off with the line clearly visible, or being dragged underwater as the gear sank.

### Line Setting and Hauling

Observation of each hook was maintained for at least 30 seconds after it left the vessel, by which time it was generally underwater and approximately 150 m astern. During line setting, if a seabird does not detect a baited hook when it enters the water, or soon after, then the baited hook is unlikely to be taken by a bird. However, when lightly weighted gear was used, baits could remain accessible to birds at 150 m astern. In order to reduce the risk of not detecting a seabird interaction with a baited hook during the setting of lightly weighted gear, distant bird activity was monitored using binoculars (7X42B Leitz trinovid), interspersed with visual scans of nearer activity.

The time and distance astern was recorded for each bait-taking attempt. Accurate estimates of the distance behind the vessel were made using observations of the vessel's fixed speed in combination with observations of the location of components of the fishing gear, such as surface floats, which are a fixed distance apart. At typical vessel speeds, for example, floats deployed every 40 seconds are 200 m astern before the next float is deployed.

By recording the time and distance astern of each bird capture it was possible to calculate when to expect that particular bird carcass to be hauled aboard. This information was used to quantify, through direct observation, the proportion of dead birds that were lost from the line once the gear was retrieved.

### Carcass Recovery

Each seabird carcass was inspected to determine the way in which it had been caught, where the manner of capture was categorized as: (i) hooked in the bill or throat (embedded) with or without entanglement in the fishing line; (ii) hooked in a location other than the bill and throat, with or without entanglement; or (iii) entangled but not hooked. Evidence of bite marks from animals such as sharks was also recorded for each carcass.

### Seabird Species and Abundance

At 15–30 minute intervals during line setting, the number of individuals of all seabird species within 500 m astern and 250 m to port and starboard were recorded, regardless of their tendency to take baits. Such counts were made 2,777 times. The presence of birds was noted during night setting as well, but not abundance. At night only portions of line were visible. Generally relatively few or no birds accompanied vessels during night sets.

### Data Analysis

Chi-square Contingency Tests (α = 0.05) determined whether the frequency of seabirds in each bait-attempt category varied significantly among fishing regions and species. The five bait-attempt categories were: successful, unsuccessful, caught, possibly caught, and unsure. The fishing regions were: Indian Ocean, Southern Ocean, Coral Sea and North-Central Pacific Ocean. Only seabird species/complex (n = 9) where observed data ≥100 individuals were analyzed to ensure sufficient replication for each bait-attempt category. Statistical tests were done using Number Crunching Statistical Analysis through *NCSS Statistical and Power Analysis* software.

## Results

### Seabird Species and Abundance

Over 25 seabird species were recorded in total from the four fishing regions (Table 1). Four groups of seabird species were too similar to always identify to the species-level reliably in the field, and so were combined into a species complex. Most seabird species were recorded from more than one fishing region, but none were found in all four regions (Table 1). Among fishing regions, the Indian and Southern Ocean seabird communities had the most similar seabird composition, sharing 17 species. Species richness was lowest in the central Pacific with three seabird species observed to follow fishing vessels, while the other three regions had 13–22 species each. The Laysan albatross (*Phoebastria immutabilis*) was the most commonly recorded seabird species, though only observed in the central Pacific (Table 1). The least abundant was the mottled petrel (*Pterodroma inexpectata*). In total, 43,193 seabirds were counted during the 15 years of this study.

**Table 1:** Number of seabirds observed by species or species complex, by fishing ground.

Family/Species	Common name	IUCN class^2^	Indian Ocean	SouthernOcean	Coral Sea	Central Pacific	Total
Diomedeidae							
*Diomedea amsterdamensis*	Wandering albatross 1	VU	105	27	5	0	137
*D. antipodensis*							
*D. dabbenena*							
*D. exulans*							
*D. epomophora*	Southern royal albatross	VU	28	0	0	0	28
*D. sanfordi*	Northern royal albatross	EN	78	0	1	0	79
*Phoebastria immutabilis*	Laysan albatross	VU	0	0	0	12,034	12,034
*P. nigripes*	Black-footed albatross	EN	0	0	0	3,546	3,546
*Phoebetria fusca*	Sooty albatross	EN	102	72	1	0	175
*P. palpebrata*	Light-mantled albatross	NT	101	383	0	0	484
*Thalassarche bulleri*	Buller's albatross	NT	1	19	66	0	86
*T. carteri*	Yellow-nosed albatross 1	EN	1,647	664	8	0	2,319
*T. chlororhynchos*							
*T. cauta*	Shy albatross	NT	354	489	179	0	1022
*T. chrysostoma*	Grey-headed albatross	VU	663	56	0	0	719
*T. melanophrys*	Black-browed albatross	EN	4,753	1,266	17	0	6,036
*T. salvini*	Salvin's albatross	VU	11	1	0	0	12
Procellariidae							
*Daption capense*	Cape petrel	LC	6,102	2,791	33	0	8,926
*Macronectes giganteus*	Giant petrel 1	NT	577	175	12	0	764
*M. halli*							
*Procellaria aequinoctialis*	White-chinned petrel	VU	1,900	31	6	0	1,937
*P. cinerea*	Grey petrel	NT	1,972	93	0	0	2,065
*P. parkinsoni*	Black petrel	VU	2	16	0	0	18
*Pterodroma inexpectata*	Mottled petrel	NT	2	0	0	0	2
*P. lessonii*	White-headed petrel	LC	17	65	0	0	82
*P. macroptera*	Great-winged petrel	LC	1,412	106	4	0	1,522
*P. mollis*	Soft-plumaged petrel	LC	46	0	0	0	46
*Puffinus carneipes*	Flesh-footed shearwater	LC	394	0	292	0	686
*P. griseus*	Shearwater 1	LC-NT	0	0	24	116	140
*P. pacificus*							
*P. tenuirostris*							
Stercorariidae							
*Catharacta antarctica*	Southern skua	LC	44	284	0	0	328

1 Species complex – a group of seabird species commonly found together interacting with fishing vessels, zztoo similar to always separate reliably in the field.

2 LC  =  least concern; NT  =  near threatened; VU  =  vulnerable; EN  =  endangered per IUCN (2009).

### Bait Taking Attempts

The proportion of seabirds recorded in some bait-taking categories differed significantly between fishing regions (χ^2^_0.05,12_ = 712; P<0.0001; Table 2). Of the 5,969 seabirds that attempted to take a bait, roughly one-third successfully removed the bait (Table 2). One hundred and seventy-six seabirds, 3% of the total, were observed being caught. Considering seabirds grouped into “possibly caught” and “unsure,” the actual number caught is probably higher.

**Table 2:** Number of seabird interactions with baited hooks by interaction category and region.

	No. Successful		No. Unsuccessful		No. Caught		No. Possibly Caught		No. Unsure		Total
Region	Observed	Expected	Observed	Expected	Observed	Expected	Observed	Expected	Observed	Expected	Observed
Indian Ocean	690	558	706	969	49	50	28	22	*223*	*97*	1,696
Southern Ocean	201	182	233	315	24	16	*24*	*7*	*70*	*32*	552
Coral Sea	73	58	62	101	*12*	*5*	*11*	*2*	19	10	177
Central Pacific	999	1,166	2,408	2,024	91	104	*16*	*47*	*30*	*203*	3,544
**Total**	1,963	1,964	3,409	3,409	176	175	79	78	342	342	5,969

Numbers are italicized when observed values differ greatly (<50% or >200%) from expected values, determined from a contingency test.

The percent of seabirds that attempted to take baits varied greatly among the 25+ species (Table 3). Some species like the northern royal albatross (*Diomedea sanfordi*), Laysan albatross, shearwaters and southern skua (*Catharacta antarctica*) were more aggressive, with >25% of individuals trying to take baits. Other species, like the wandering albatross (*Diomedea amsterdamensis*), sooty albatross (*Phoebetria fusca*), cape petrel (*Daption capense*) and white-headed petrel (*P. lessonii*) rarely attempted to take baits even though they were commonly recorded around fishing vessels. The cape petrel was the second most common seabird in this study, for example, but only 20 of the 8,926 individuals counted were seen trying to take baits.

**Table 3:** Number of seabirds recorded in five baited hook interaction categories by seabird species/species complex.

Seabird	Successful	Unsuccessful	Caught	Possibly caught	Unsure	Total	% of abundance
Wandering albatross 1	5	2	0	0	2	9	6.6
Southern royal albatross	0	0	0	0	0	0	0.0
Northern royal albatross	7	4	3	1	5	20	25.3
Laysan albatross	911	2,043	75	8	22	3,059	25.4
Black-footed albatross	73	271	16	8	1	369	10.4
Sooty albatross	3	7	0	0	0	10	5.7
Light-mantled sooty albatross	18	32	2	4	9	65	13.4
Buller's albatross	0	4	0	0	0	4	4.7
Yellow-nosed albatross 1	54	123	2	6	8	193	8.3
Shy albatross	73	26	7	7	15	128	12.5
Grey-headed albatross	19	60	8	2	1	90	12.5
Black-browed albatross	228	393	40	31	54	736	12.2
Salvin's albatross	3	0	0	0	0	3	25.0
Cape petrel	16	3	0	1	0	20	0.2
Giant petrel 1	24	14	6	3	11	58	7.6
White-chinned petrel	62	65	0	1	80	208	10.7
Grey petrel	51	45	2	0	8	106	5.1
Black petrel	0	1	0	0	0	1	5.6
Mottled petrel	0	0	0	0	0	0	0.0
White-headed petrel	0	0	0	0	0	0	0.0
Great-winged petrel	281	70	0	0	8	359	23.6
Soft-plumaged petrel	1	0	0	0	0	1	2.2
Flesh-footed shearwater	76	110	10	12	10	218	31.8
Shearwater 1	1	38	0	0	6	45	32.1
Southern skua	37	40	3	2	2	84	25.6
Unknown	5	2	2	3	99	111	

Also shown for each species, is the percent of individuals interacting with the bait compared to its total abundance.

1 Species complex.

Seabird species that commonly attempted to take baits differed in their likelihood of success or of getting caught (χ^2^_0.05,32_ = 1460; P<0.0001) (Table 4). This indicates that some species, such as the great-winged petrel (*P. macroptera*) are very successful at taking baits without getting caught, while other species like the black-browed (*Thalassarche melanophrys*) and black-footed albatrosses (*P. nigripes*) get caught more often than expected (Table 4).

**Table 4:** Comparison of observed vs. expected (determined from a contingency test) values for seabird species or species complexes where > 100 individuals attempted to take baits.

	No. Successful		No. Unsuccessful		No. Caught		No. Possibly caught		No. Unsure	
Seabird	Observed	Expected	Observed	Expected	Observed	Expected	Observed	Expected	Observed	Expected
Laysan Albatross	911	1,027	2,043	1,787	75	86	*8*	*42*	*22*	*117*
Black-footed Albatross	73	124	271	216	16	10	8	5	*1*	*14*
Yellow-nosed Albatross 1	54	65	123	113	*2*	*5*	6	3	8	7
Shy Albatross	73	43	*26*	*75*	7	4	7	2	*15*	*5*
Black-browed Albatross	228	251	393	436	40	21	*31*	*10*	54	29
White-chinned petrel	62	70	65	121	*0*	*6*	*1*	*3*	*80*	*8*
Grey petrel	51	36	45	62	2	3	*0*	*1*	8	4
Great-winged petrel	*281*	*121*	*70*	*210*	*0*	*10*	*0*	*5*	8	14
Flesh-footed shearwater	76	73	110	127	10	6	*12*	*3*	10	8

Numbers are italicized when observed values differ greatly (<50% or >200%) from expected values.

1 Species complex.

### Seabird Carcasses

Of the 176 seabirds that were observed getting caught (Table 2), only 85 carcasses (48%) were retrieved. Thus, more than 50% of seabirds observed caught during setting were not attached to the gear when retrieved. Among fishing regions, the percent of caught birds observed on the set that were subsequently retrieved during hauling was similar for the Indian Ocean, Southern Ocean and Central Pacific, ranging from 45–55%, but in the Coral Sea 9 of the 12 caught birds were hauled aboard.

For the 553 seabird interactions recorded as “possibly caught” or as “unsure”, 42 carcasses were retrieved. For the 5,367 seabird interactions recorded as “successful” or as “unsuccessful”, and thus considered “not caught,” 5 carcasses were retrieved, indicating a very low mistake rate: only 0.09% of the birds observed to have successfully removed bait from a hook without being caught and birds observed to have been unsuccessful in their attempts to remove bait from hooks and also without being caught were captured as documented by being retrieved dead on the haul.

Most (>60%) of the retrieved carcasses were Laysan or black-browed albatrosses, the two species that were most commonly recorded “caught” (Table 3). The majority of the 132 seabird carcasses were hooked in the bill or throat (79%), with the remainder hooked externally or tangled in the fishing line. Five carcasses showed bite marks.

## Discussion

Through analyses of data collected from four regions over 15 years, our findings document that previously reported estimates of seabird bycatch in pelagic longline fisheries, estimated through observations made during gear retrieval, are likely half of actual mortality levels, calling for revised data collection protocols and adjustments to seabird population model inputs. The large difference between the total numbers of seabirds observed caught during setting and the number of carcasses retrieved during gear hauling clearly shows that using only data collected during the haul grossly underestimates seabird bycatch. This is consistent with previous findings [8], [13], [19]–[20], [25], [27]. This study suggests that pelagic longline fishing catches and kills twice the number of seabirds previously thought, and is a substantially larger threat to pelagic seabirds than previously understood.

Observations for estimates of seabird removal between the set and haul here for the North-Central Pacific region were consistent but slightly higher than findings by Gilman et al. [20], [25] for the Hawaii longline fishery. A larger sample size was employed in the present study. The cause of the difference in findings is not clear.

Several factors may explain why hooked seabirds are not retrieved during hauling. The low incidence (4%) of seabird carcasses that had bite marks suggests that scavenging by sharks, if this mechanisms is causing a large proportion of the observed seabird removal during the gear soak, generally removes the entire bird. Blue (*Prionace glauca*) and mako (*Isurus oxyrinchus*) sharks are often large in size and are commonly caught as bycatch on pelagic longlines [28]–[30]. Seabirds could also be torn off the hook during line setting or hauling from mechanical action [19]. Some seabirds observed caught during the set may have eventually dislodged the hook, or untangled from the line, and escaped [25]. This might occur, however, once the caught bird is far astern where observations are limited. Also, crew have been observed to remove captured seabirds from branchlines during hauling operations such that observers would fail to detect the captured bird during routine monitoring during hauling [13], but this was not a factor in the current study due to the observation methods employed by the researcher.

Other aspects of seabird-fishery interactions further affect the accuracy of estimates of total seabird mortality. For example, hooks discarded in offal (spent bait and fish parts) can result in seabird mortality: Weimerskirch & Jouventin [31] observed wandering albatrosses on their nests injured from hook wounds. Longline vessels discarding hooks in offal, although mostly a feature of demersal longline fisheries, and crew cutting free birds caught during hauling are two sources of these hooks [18]. Mortality of one albatross of a breeding pair usually results in chick mortality by starvation, and the remaining adult albatross partner will take several years before mating again, further reducing reproductive output [18], [32].

There are numerous factors that likely have a significant effect on seabird catch rates as well as on seabird falloff during the soak, such as hook and bait size and type, the time interval difference between the capture time of the bird and the haul time, the depth that hooks are set, branchline length, hauling rate, current strength at fishing grounds, and abundance of sharks and other predators. These factors vary between vessels in a fleet, and between fleets.

Species-specific behavior by pelagic seabirds affects their vulnerability to capture in longline fisheries. Seabird species varied greatly in their interactions with baited hooks, with some species like the Laysan albatross and Southern Skua often attempting to take baits while other species like the Southern royal albatross (*Diomedea epomophora*) and white-headed petrel (*Pterodroma lessonii*) did not take any baits. In addition, seabird species differed in their success in removing baits, with some species like the great-winged petrel (*P. macroptera*) twice as likely to successfully remove baits as other species. Black-browed albatrosses, in contrast, were caught more often than expected. Several factors help explain differences among species. Bold behavior, for example, increases the likelihood of being caught. Species abundance plays a role, where the more abundant species like the Laysan and black-browed albatrosses often attempting to take baits with correspondingly higher numbers getting caught. In contrast, some abundant species, like the cape petrel, rarely took baits. Northern royal albatrosses lack aerial agility and do not submerge totally so will generally only take baits that are within reach of the surface. Although often out-maneuvered by other species, they aggressively compete for baits already seized by another species, increasing their chance of being caught. Over many weeks of fishing, certain individuals recognizable by distinctive plumage features were observed to disassociate themselves from fishing activity altogether, despite remaining in the vicinity of the fishing vessel, perhaps altering their behavior as a result of experiencing capture or near-capture.

If observations for this study had been made in regions where there was a greater frequency of interactions between deep diving seabird species and albatrosses, it would not have been possible to obtain reliable estimates of bird captures during setting because seabird capture events would have occurred at a greater distance astern, potentially outside of the researcher's field of vision. For example, the deep-diving flesh-footed shearwater (*Puffinus carneipes*), one of the two most often caught species in the Australian pelagic longline fishery, can reach baits to a depth of 20 m, when the baited hooks are far astern. As a result, these deep-divers are caught on baited hooks or bring baited hooks to the surface also far astern, where they then become available to larger species of albatrosses, petrels and skuas [33].

The vast majority of seabirds that attempted to take baits were not caught, with many birds successfully removing the bait from the hook. Added to the number of caught birds, this represents a loss of at least 2,139 baits. This is only a small fraction (<0.5%), however, of the total number of baited hooks set. This small loss of fishing opportunity and thus low economic impact is likely a key reason why fishers are not highly motivated to reduce seabird access to baited hooks.

This study highlights the need for data collection protocols by onboard observers to include recording seabird captures during line setting in addition to observing and recording the smaller proportion of total captures that occur during gear retrieval. Available estimates of seabird bycatch in longline fisheries have been based only on observations during hauling.

This study shows that roughly half of birds caught during pelagic longline setting are not retrieved when the gear is retrieved, consistent with results from past studies [8], [13], [20], [25]. In addition to the need to account for all seabirds caught during longline fishing operations, to provide a more precise input for population models, estimates of seabird mortality in longline fisheries require adjusting to also account for delayed mortality of a proportion of seabirds caught but freed from gear, and mortality caused by hooks discarded in offal [19].
